# Making Meaningful Improvements in Pharmacotherapy and Medication Management for Children and Youth—A Modest Proposal

**DOI:** 10.3390/children6100111

**Published:** 2019-10-09

**Authors:** Richard H. Parrish, Johannes van den Anker, Sandra Benavides

**Affiliations:** 1 St. Christopher’s Hospital for Children, 160 E. Erie Avenue, Philadelphia, PA 19134, USA; 2 Virginia Commonwealth University, School of Pharmacy, Richmond, VA 23298, USA; 3 Universitäts-Kinderspital beider Basel (UKBB), Spitalstrasse 33, CH-4031 Basel, Switzerland; JohannesN.VandenAnker@ukbb.ch; 4 Children’s National Health System, 111 Michigan Avenue, Washington, DC 20010, USA; 5 Erasmus Medical Center—Sophia Children’s Hospital, s-Gravendijkwal 230, 3015 CE Rotterdam, The Netherlands; 6 PRIME Education, Miami, FL 33169, USA; sandra.b.caballero@gmail.com

**Keywords:** children, youth, clinical pharmacy, pediatrics, medication management, medical complexity

The Special Issue, “Development of a National Pediatric Pharmacotherapy Collaborative Practice Network,” has illuminated the vital global need for better care coordination and interprofessional collaboration in pharmacotherapy and medication management of children with medical complexity and special healthcare needs (CSHCN-CMC). From a population health perspective, we identified how coordination and outcomes might be improved from both practice and research perspectives, and what tools exist to assist clinical pharmacists and pediatricians to build more durable and sustainable comprehensive medication management practices around the world [1,2]. The work of the Professional Compounding Centers of America (PCCA) group [3] and ‘GOLDILOKs’ (Genome and Ontogeny-Linked Dose Individualization and cLinical Optimization for KidS) Clinic [4] illustrated personalized approaches to identifying, understanding, and responding to these patients’ unique drug therapy needs with evidence-based compounded formulations and genome-informed care planning. Ohio’s Partners for Kids Accountable Care Organization (ACO) provided a comprehensive, systems-based program for including clinical pharmacists to address inefficiencies in care coordination and delivery, especially in children with asthma and behavioral health needs [5]. The development and inclusion of clinical pharmacists within maternal child health strategies of the Health Resources and Services Administration’s Pediatric Pulmonary Center in Arizona fostered improvements in community-based, family-centered healthcare for children with chronic respiratory diseases [6]. Clearly, these approaches portend increased responsiveness to unmet drug-related needs at individual and system levels of analysis.

What’s next? To continue to build on these successes, the following recommendations are offered:

1. Creation of interprofessional communities of practice and research that share tacit knowledge through various synchronous and asynchronous delivery media to promote point-of-care collaboration and coordination;
2. Application of administrative leadership and vision that promotes an environment of innovation through sustainable provider-patient-payer partnerships and outcome-driven system transformation;
3. Adoption and expansion of patient engagement functionalities that align with the 21st Century Cures Act and the World Health Organization’s Convention on the Rights of the Child for increasing choice, access, equity, and quality in health care.

We believe this modest proposal will generate meaningful improvements that assure all children receive optimal benefit from pharmacotherapies. We thank the authors and readers of these manuscripts, and hope that this dialogue will continue to build more durable and sustainable care coordination systems locally and beyond.
